# Conceptualizing the dynamics of workplace stress: a systems-based study of nursing aides

**DOI:** 10.1186/s12913-016-1955-8

**Published:** 2017-01-05

**Authors:** Arif Jetha, Laura Kernan, Alicia Kurowski, Pro-Care Research Team

**Affiliations:** 1 Department of Work Environment, University of Massachusetts Lowell, Lowell, MA USA; 2 Institute for Work & Health, 481 University Avenue, Suite 800, Toronto, Ontario M5G2E9 Canada; 3 Centers for Disability Research and Behavioral Science, Liberty Mutual Research Institute for Safety, Hopkinton, MA USA; 4 DMA Health Strategies, Lexington, MA USA

**Keywords:** Workplace stress, System dynamics, Nursing aides

## Abstract

**Background:**

Workplace stress is a complex phenomenon that may often be dynamic and evolving over time. Traditional linear modeling does not allow representation of recursive feedback loops among the implicated factors. The objective of this study was to develop a multidimensional system dynamics model (SDM) of workplace stress among nursing aides and conduct simulations to illustrate how changes in psychosocial perceptions and workplace factors might influence workplace stress over time.

**Methods:**

Eight key informants with prior experience in a large study of US nursing home workers participated in model building. Participants brainstormed the range of components related to workplace stress. Components were grouped together based on common themes and translated into feedback loops. The SDM was parameterized through key informant insight on the shape and magnitude of the relationship between model components. Model construction was also supported utilizing survey data collected as part of the larger study. All data was entered into the software program, Vensim. Simulations were conducted to examine how adaptations to model components would influence workplace stress.

**Results:**

The SDM included perceptions of organizational conditions (e.g., job demands and job control), workplace social support (i.e., managerial and coworker social support), workplace safety, and demands outside of work (i.e. work-family conflict). Each component was part of a reinforcing feedback loop. Simulations exhibited that scenarios with increasing job control and decreasing job demands led to a decline in workplace stress. Within the context of the system, the effects of workplace social support, workplace safety, and work-family conflict were relatively minor.

**Conclusion:**

SDM methodology offers a unique perspective for researchers and practitioners to view workplace stress as a dynamic process. The portrayal of multiple recursive feedback loops can guide the development of policies and programs within complex organizational contexts with attention both to interactions among causes and avoidance of adverse unintended consequences. While additional research is needed to further test the modeling approach, findings might underscore the need to direct workplace interventions towards changing organizational conditions for nursing aides.

## Background

Workplace stress is a complex and dynamic experience often reported by workers in health care settings. Nursing aides, in particular, report a number of interrelated psychological and physical workplace and non-workplace factors that may contribute to variable stress responses [1, 2]. To date few studies have examined complexity inherent in workplace stress in health care settings, especially among nursing aides. While currently used models in the field of occupational health and safety have uncovered individual, psychosocial, and organizational factors associated with workplace stress, they may not examine the dynamic feedback relationship between these factors that can be a source of complexity. Through the lens of system dynamics modeling, we take a sociotechnical systems perspective to conceptualize perceptions of workplace stress among nursing aides.

Workplace stress can be operationalized as both an outcome of factors inside and outside of work, and can be a determinant to health and quality of life and performance of work roles. This may be especially the case for nursing aides who report challenging working conditions that may include little assistance with job tasks, high psychological and emotional demands, few opportunities for decision-making, and poor safety climate [3–5]. While little research has focused on workplace stress among nursing aides, several studies of allied health professionals have sought to examine the association between workplace factors and stress. These studies find that work factors including job demands and lack of time, low managerial support, patient aggression, non-standardized working conditions (e.g., shift work) and scheduling unpredictability were related to high perceptions of workplace stress [6–12]. At the same time, workplace stress can also be conceptualized as a determinant to health and work outcomes. A number of studies find that within healthcare settings, workplace stress can be associated with poor physical [13, 14] and psychological health [15–19], and difficulties with job performance including lower job motivation [20], reduced job satisfaction [14, 15, 20], greater intention to leave work [21], higher job turnover [14], absenteeism [22], and presenteeism [22]. Based on existing research, workplace stress could also be conceptualized within the framework of a dynamic feedback loop, which could reflect its role as both an outcome and determinant. No studies to our knowledge have examined workplace stress through the lens of a feedback model.

The predominant model examining and measuring workplace stress is the Job Demand-Control model (JDC) [1, 23, 24]. The JDC conceptualizes stress (referred to as job strain) as a function of a worker’s perceived job demands and job control. The model posits that higher job demands coupled with lower job control contributes to higher levels of job strain. Workers with higher levels of job strain are theorized to have a greater risk of health issues [1, 23, 25]. In a more recent iteration, co-worker and supervisor support were added as determinants to job strain [26]. The JDC has successfully isolated key factors that influence workplace stress, and has motivated a number of interventions including those that build individual coping strategies [27], address co-worker and managerial support, and minimize physical and psychological job loads [28]. At the same time, the JDC has traditionally examined job demands, job control, and workplace support as having a constant and additive effect on job strain and may not account for its dynamic feedback relationship between influential variables [29]. To advance our understanding of workplace stress, a sociotechnical systems-based view of workplace stress can be taken [29]. The need for a systems based approach has been acknowledged by Robert Karasek, author of the JDC, who suggested that: “…there may be many causes which “accumulate” to contribute to a single effect; a single cause (“stressor”) may have many effects; or effects which occur only after significant time delays” (1998) [30].

A sociotechnical perspective considers organizations as adaptive systems made up of interdependent personal, social, technical, and organizational components that interact with one another in non-linear ways [31, 32]. System dynamics modeling (SDM) is one specific sociotechnical systems-based methodology [31]. SDM was originally designed for understanding complex problems in business and engineering. More recently it has been applied to the field of public health [33–35] where it has been useful in detecting system components amenable to intervention. When applied to understanding workplace stress, SDM has several key benefits for researchers and practitioners. First, it provides a visual depiction of relationships of components using feedback loops that have amplifying (e.g. action generating) or balancing (e.g. maintaining status quo or dampening) effects on the system [31]. SDM also provides a practice-based simulation tool to test dynamic hypotheses and determine the system-wide impacts of modifying different components. The simulation tool permits researchers to show practitioners the likely outcomes of policies or programs and engage stakeholders in critical discussions on stress reduction and health promotion planning [36]. As a practical tool, SDM can also enable simulations that help practitioners and program planners understand how interventions might impact workplace stress over time. The overall objective of the current study is to apply a sociotechnical system thinking perspective to address dynamics of workplace stress among nursing aides, and examine the multiple influential factors using SDM methodology. Findings from this study will offer a unique view of workplace stress and motivate system-focused strategies.

## Methods

A multi-staged model building methodology based on key informant insights was conducted [37]. Model development was set within the context of a larger examination of factors associated with the physical and mental health of nursing aides in nursing home facilities in the eastern United States, entitled “Promoting Physical and Mental Health of Caregivers through Trans-disciplinary Intervention (Pro-Care) [38, 39]. The study was approved by the University of Massachusetts Lowell Institutional Review Board (Protocol #12-056).

### Model construction process

Eight key informants were recruited to participate in model building. Key informants were chosen because of their exposure to the personal, psychosocial, and organizational conditions of nursing aides. Each were members of the Pro-Care investigative team for an average of 6 years and conducted field work directly with nursing aides in nursing home facilities. To participate, all key informants had to have in-depth knowledge of health, workplace, and personal experiences of nursing aides that they were willing to share with others. Prior to model building, each participant was given a short tutorial on sociotechnical systems to facilitate broader thinking regarding workplace stress. All key informants then engaged in four model-building sessions that lasted 60 to 90 minutes and consented to having their insights incorporated into the model. An external model builder facilitated discussions to elicit the structure and process of the workplace stress system, while concurrently translating conversations into visual mapping of the SDM [37].

In the first session, key informants discussed the scope of the model and established model boundaries at the organizational level. As part of this initial conversation, informants talked about workplace stress broadly as a major determinant of health and well-being of nursing aides. The second model building session involved brainstorming the range of influential components within an organizational system boundary. The model boundary was set at the organizational level to focus sessions, and allow for comparisons between nursing home sites in different community contexts [35]. When participants discussed an influential component, follow-up questions were asked about how the component might influence workplace stress. Components were ultimately clustered into common themes and translated into feedback loops by the model-builder. During the initial sessions, participants were also asked to think about whether components that made up each feedback loop should be broad or fine-grained. As a second model building strategy, participants were presented with other workplace stress models and asked to discuss whether other components should be included in a model. All discussions were incorporated into a representative SDM [37].

Following the first two key informant sessions, construction of the model began with an examination of the relationship between components identified by the key informants using Pro-Care data. Further model building was conducted with a subgroup of three key informants. These subgroup sessions aimed at critically discussing and confirming the structure of each feedback loop. The refined SDM generated through the subgroup sessions was shared with the larger group of key informants who were asked for their level of agreement regarding model accuracy in representing the factors associated with workplace stress. In cases where there was disagreement, further discussions were conducted among the subgroup, and the structure of the model was refined and fed back to the larger group. This iterative process continued until agreement on the description of each feedback loop was reached. After several critical discussions, a final feedback structure of the model was generated and entered into the system dynamics modeling software program, Vensim [40].

### Parameterization and simulation

After determining the feedback structure, the SDM was parameterized. First, baseline mean values for each component were entered into Vensim as parameter values that reflected initial model conditions. These values were extracted from a Pro-Care survey administered to nursing aides in 24 sites from 2012 to 2013 (*n* = 950). The Pro-Care survey was administered biannually to nursing home staff and measures a range of concepts including individual characteristics, psychosocial perceptions, and workplace conditions. In our model, job strain was utilized as a proxy for perceived workplace stress [41, 42]. Job strain was measured using the Job Content Questionnaire and was computed using the difference in scores from job demands and job control items [42].

To determine the nature of the feedback relationship between model components over time, a reference mode exercise was also undertaken [35]. Key informants were presented with axes that included a system component on the horizontal axis, and an outcome of the feedback loop on the vertical axis. Participants were asked to estimate the direction, shape, and magnitude of the relationships between the variables on each axis, forming a reference mode [35]. Reference modes were created to examine changes to workplace stress over a 10-week time horizon, noted by key informants as being the duration of time required to capture the workplace stress response. Similar to the model building methodology, reference mode disagreements were iteratively discussed between key informants to reach consensus. Using the final shape of the reference modes, which reflected the relationships between variables, separate differential equations were generated and entered into the Vensim software program [37].

Lastly, using the model entered into Vensim, simulations were conducted to test how decreasing or increasing one or more components (e.g. workplace conditions or demands outside of work) in the model influenced workplace stress over time. Using the simulation model, model sensitivity tests were also conducted. First, component values were set to extreme conditions to determine if changes to workplace stress occurred as expected [37]. Second, simulation results were compared to correlational data collected by the Pro-Care research team. In cases where large discrepancies existed between the reference mode and findings from the survey data, the model builder and key informants discussed the discrepancy and refined the model relationships.

## Results

### Description of model structure

**Fig. 1:**
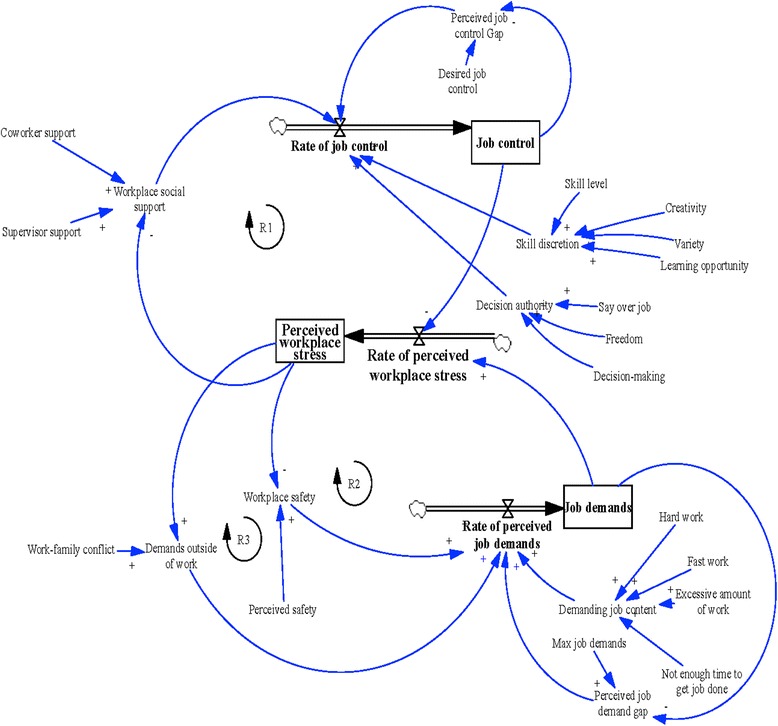
System dynamics model of components related to workplace stress. Rectangle box indicates stock variable that accumulates or depletes over time; Thick arrows indicates a flow variable which refers to the rate of change in the stock over time; ‘+’ = A positive relationship which indicates that components change in the same direction; ‘-‘ = A negative relationship indicates that components change in different directions

The SDM presented in Fig. 1 represents the causal loop structure of the workplace stress system that emerged from key informant model building sessions. Not surprisingly, the SDM was multidimensional and included several factors including perceptions of organizational conditions; job demands (e.g., difficulty and pace of work, time available to perform tasks), job control (e.g., discretion over job, freedom, learning opportunities), organizational social factors (i.e., managerial and coworker social support), and demands outside of work (i.e. work-family conflict). Participants also included workplace safety as a broader system component to capture the various risk exposures nursing aides may experience within their jobs. The polarity between related components was established during model building and is depicted in Fig. 1 as positive (‘+’ denotes that components change in the same direction) or negative (‘-’ denotes that components change in opposing directions).

As exhibited in the SDM, workplace stress was the primary stock (level of outcome) and flow (rate of change of outcome). Aligning with previous workplace stress models, key informants indicated that job demands and job control represented two secondary stocks and flows that impacted workplace stress. Indicated by the positive polarity ‘+’, an increase in job demands led to an increase in the flow of workplace stress. As discussed in model building, several components amplified the stock and flow of job demands including psychological demands (i.e., speed and difficulty of work, available time to perform jobs, amount of work) (+), workplace safety (+), and work-family conflict (+). Participants reported that job control was the second stock and flow that impacted workplace stress. Findings from model building showed that job control had an opposing effect. An increase in job control would dampen workplace stress (−). In the model building process, key informants discussed several components that impacted job control including skill discretion (i.e., skill level, creativity, variety, and learning opportunities), decision authority (e.g., say over jobs, and decision making capacity) (+), and workplace support (+).

Interestingly, through model building, three reinforcing feedback loops that generated action in the workplace stress system were described. In the first reinforcing feedback loop (R1), workplace social support was positively linked with job control (+); job control was negatively linked with workplace stress (−); and a negative link existed between workplace stress and workplace social support (−). The second reinforcing feedback loop involved workplace safety (R2). Greater workplace safety was related to lower job demands (−); job demands were positively linked with workplace stress (+); and workplace stress was negatively linked with workplace safety (−). As reflected in the last reinforcing feedback loop (R3), greater conflict between work and family contributed to more job demands (+); job demands were positively linked with workplace stress (+); and workplace stress was positively linked with work-family conflict (+).

### Simulation scenarios

**Fig. 2:**
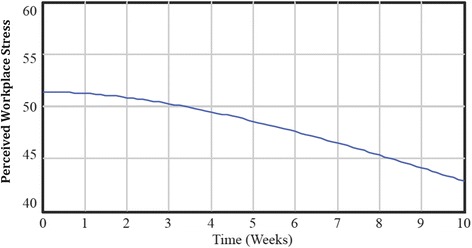
Findings from system dynamics model simulation base case

Using the key informant-designed SDM, simulation scenarios were conducted to determine how changes to perceptions of organizational conditions, organizational social support, workplace safety, and work-family conflict could impact workplace stress within the nursing homes in which the model was based. System behavior was simulated over the 10-week time horizon set by participants. Simulations provide important practice-based insight into how adapting model components could impact workplace stress and uncover potential points of intervention [4]. For interpretability, model simulations were converted into a workplace stress score. All findings were compared to a base case scenario which was simulated using initial values, taking into account the feedback loops discussed earlier. As indicated in the base case model simulation (Fig. 2), workplace stress declined only slightly over a ten-week period; 51.0 (0 weeks) to 48.9 (5 weeks) to 42.7 (10 weeks).

**Fig. 3:**
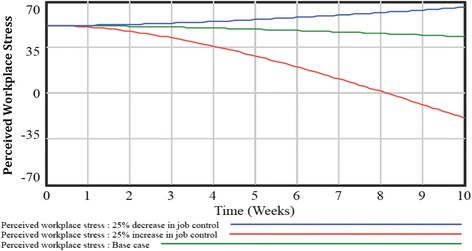
Findings from system dynamics model simulation scenarios examining changes in job control

**Fig. 4:**
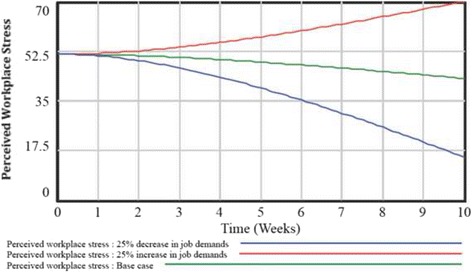
Findings from system dynamics model simulation examining job demand scenarios

Next, simulations were conducted to examine how changes to model components impacted workplace stress. First, simulations were conducted to compare job control base case simulation to increased (25% increase) and decreased (25% decrease) scenarios. As reflected in Fig. 3, increasing job control was associated with an exponential decrease in workplace stress from 51.0 (baseline) to 27.9 (five weeks) to −19.62 (ten weeks). Conversely, the decreased job control scenario was associated with an exponential increase in workplace stress from 51.0 (baseline) to 55.9 (five weeks) to 65.3 (ten weeks). The impact of job demands on workplace stress was the second simulation conducted (Fig. 4). A decreased (25% decrease) and increased (25% increase) job demand scenario was compared to the base case. Simulation findings showed that decreasing job demands in the workplace was associated with an exponential decrease in workplace stress from 51.0 (base case) to 39.3 (five weeks) to 14.8 (ten weeks). Conversely, the increasing job demands scenario was associated with an exponential increase in workplace stress from 51.0 (base case) to 57.5 (five weeks) to 70 (ten weeks).

**Fig. 5:**
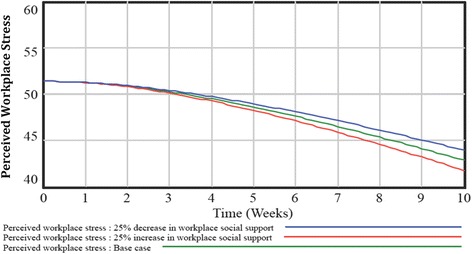
Findings from system dynamics model simulation scenarios examining changes in workplace social support

**Fig. 6:**
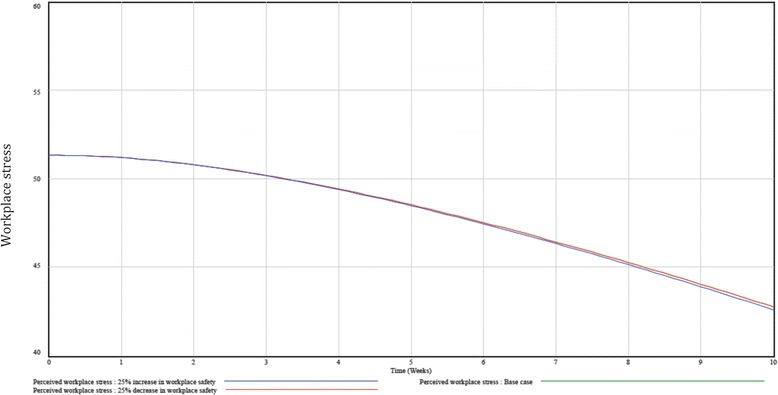
Findings from system dynamics model simulation scenarios examining changes in workplace safety

**Fig. 7:**
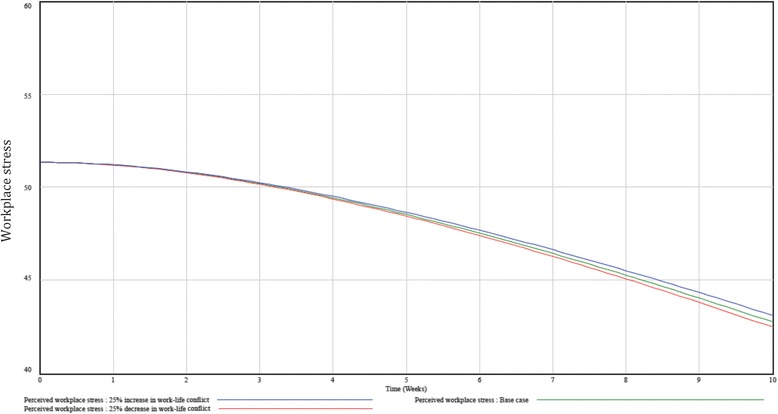
Findings from system dynamics model simulation scenarios examining changes in work-family conflict

The next set of simulations examined how changes to the remaining system components (i.e., workplace social support, safety, and demands outside of work) would impact workplace stress. The base case was compared to an increased workplace social support scenario (co-worker and managerial support increased by 25%), and a decreased workplace social support scenario (co-worker and managerial support decreased by 25%). Interestingly, both simulation scenarios followed a similar declining trajectory (Fig. 5). However, the increased social support scenario was associated with a slightly greater rate of decline in workplace stress from 51.0 (baseline) to 48.2 (five weeks) to 41.6 (ten weeks). In comparison, a decrease in workplace social support was associated with a marginally reduced rate of change in workplace stress ranging from 51.0 (baseline) to 48.9 (five weeks) to 43.8 (ten weeks). As described in Fig. 6 in Appendix 1 and Fig. 7 in Appendix 2, simulations conducted with workplace safety and work-family conflict had a similar marginal impact on workplace stress.

## Discussion

This study takes a dynamic systems-level view of workplace stress among nursing aides. The model was developed through key informant insights and consisted of multiple feedback loops containing a diverse range of components including perceptions of organizational conditions, workplace social support, workplace safety, and work-family conflict. All model-building simulations incorporate parameter values from a survey of nursing aides and initial model validity was conducted. Consistent with previous research, within the context of the SDM, simulations showed the importance of reducing job demands and increasing job control to improve long-term experiences of workplace stress among nursing aides. A systems thinking perspective provides a useful tool for practitioners, such as nursing home administrators and workplace health personnel, who are tasked with designing policies or programs to minimize stress among nursing aides.

The SDM offered a novel view of workplace stress that captured a broader set of components and their feedback relationships. Consistent with previous models of workplace stress, key informants discussed the day-to-day impacts of organizational conditions (e.g., job demands and job control) as important components in the model, along with workplace social support and workplace safety, and demands outside of work (i.e., work-family conflict). Feedback loops consisting of model components were developed that had distinct amplifying effects on workplace stress. Feedback loops illustrate the cyclical nature of workplace stress, which is conceptualized as both an outcome and determinant of conditions both inside and outside of the workplace. Based on the perspective provided by our model, workplace stress may be addressed by designing workplace interventions that account for the multiple factors within an organizational system, and programs that may disrupt reinforcing feedback relationships between model components.

By building a systems-based simulation model, this study aimed to expand on classic workplace stress models. Interestingly, the base case scenario suggested that over the 10-week period workplace stress was relatively constant, and underscores the need to develop primary and secondary workplace interventions for nursing aides. As shown in the subsequent simulation scenarios, improvement in the organizational conditions will help to treat workplace stress. Small organizational changes that could enable nursing aides to have more control and experience fewer demands were related to a decline in workplace stress over the simulation period. Conversely, policy changes that may increase demands or reduce control can have an opposing effect, and result in a reinforcement of workplace stress over time. It is important to acknowledge that organizational policies, low institutional resources, restrictions to the number and type of tasks a nursing aide performs can limit significant changes in the clinical workplace settings. At the same time, our study suggests that potentially minor changes to perceptions of organizational conditions can generate conditions that weaken the stock and flow of workplace stress.

Within the context of the nursing homes and nursing aides in which key informants had insights, workplace social support, workplace safety and demands outside of work (e.g., work-family conflict) were important components in the SDM. In contrast to findings from previous studies [14, 43–45], systems-based simulations indicated that modifying these components did not have a large impact on the stock and flow of workplace stress. This could suggest a potential model bias due to the fact that the informants were all research personnel; inputs from nursing home workers, supervisors, or managers might have affected the components included in the model and their impact on workplace stress. At the same time, findings could further indicate that within the perspective of the broader system, factors such as perceptions of job control and demand play a dominant role in workplace stress. Additional research and modeling is required to further examine these factors on the dynamics of workplace stress.

There were several benefits related to the model building methodology. By participating in model building sessions, participants were able to gain a greater awareness of the complexity of stress within the organizations with which they were working and develop an understanding of how to devise and implement changes within the dynamic system. Through model simulations, decision-makers can also test hypotheses, examine how various interventions impact workplace stress, and better understand why interventions may not have intended outcomes. Based on the model we produced, findings point towards changing organizational conditions rather than on workplace social support, workplace safety, and work-family balance to improve perception of workplace stress. Ultimately, insights gained from the model building process can be valuable to engage workplace health promoters and nursing home administrators with ways that they can direct scarce resources to have the greatest impact on minimizing workplace stress.

There are methodological strengths and weaknesses worth noting. Our model was built using insights from key informants who had in-depth observational knowledge of the nursing aides’ personal and work-related experiences and were able to think about various components from a sociotechnical systems perspective. This knowledge was incorporated into the feedback loops that made up the model and enabled us to test the feasibility of SDM in examining workplace stress among key informants. Additionally, our model of workplace stress was data-driven and incorporated parameter values from a large survey of nursing home staff. We utilized results from a multi-center longitudinal study of nursing homes to establish initial model conditions, and as a way to examine preliminary model validity. Although our model was developed within the context of specific US nursing homes, it may have some applicability to other settings. While specific physical and psychological exposures of nursing aides in nursing homes might differ from other clinical settings, features of the job and perceptions of workplace stress may be similar to other allied health professionals. Moving forward, it is suggested that additional research applying the model building methodology to workplace stress among a broader range of healthcare workplaces is required to understand the generalizability and the reliability of the approach.

There are also several limitations. First, learning to view and express workplace stress as a system of interrelated components can be challenging. Through a series of probing questions, the model-builder encouraged team members to think more holistically about workplace stress [37]. While we utilized a multi-staged iterative model building approach, there may have been other important factors that could have been included in the model. While model building based on key informant insights provided support for the feasibility of SDM methodology, the model could suffer from potential biases. To supplement key informant insights and build upon the current model, it is recommended that future model building include nursing aides and other stakeholder groups, each of whom may have a different perspective [46, 47], and representation from different types of long-term care facilities to uncover a greater range of components related to workplace stress in this sector [11, 48].

## Conclusions

Workplace stress reported by nursing aides occurs within the context of a dynamic and complex organizational system. Through the application of system dynamics modeling methodology, key informants identified a range of interrelated variables associated with workplace stress among nursing aides. The constructed model depicted the perceived importance of job control and job demands to long-term workplace stress. Model simulations suggested that workplace social support, workplace safety, and work-family conflict appeared to be less integral within the context of the broader system. Based on the model designed in this study, there is a need to direct workplace interventions towards changing organizational conditions. Findings underscore the importance of conceptualizing the system as a whole when designing policies and programs to reduce stress in the workplace. The SDM model can be a valuable educational tool for managers by illustrating dynamics over time and enabling assessment of “what if” scenarios from changes in programs and policies; the results of this work will be used to inform future dissemination products and strategies.

## Abbreviations

JDC: Job demand-control model
PRO-CARE: Promoting physical and mental health of caregivers through trans-disciplinary intervention (pro-care)
SDM: System dynamics model
